# Cost effectiveness of a mail-delivered individually tailored physical activity intervention for Latinas vs. a mailed contact control

**DOI:** 10.1186/s12966-015-0302-5

**Published:** 2015-11-11

**Authors:** Britta Larsen, Todd Gilmer, Dori Pekmezi, Melissa A. Napolitano, Bess H. Marcus

**Affiliations:** 1 Department of Family Medicine and Public Health, University of California, 9500 Gilman Dr, San Diego, CA 92093-0628 USA; 2 Department of Health Behavior, School of Public Health at University of Alabama, Birmingham, USA; 3 Departments of Prevention and Community Health & Exercise and Nutrition Sciences, The George Washington University School of Public Health, Washington, USA

## Abstract

**Background:**

Physical inactivity is high in Latinas, as are chronic health conditions. There is a need for physical activity (PA) interventions that are not only effective but have potential for cost-effective widespread dissemination. The purpose of this paper was to assess the costs and cost effectiveness of a Spanish-language print-based mail-delivered PA intervention that was linguistically and culturally adapted for Latinas.

**Methods:**

Adult Latinas (*N* = 266) were randomly assigned to receive mail-delivered individually tailored intervention materials or wellness information mailed on the same schedule (control). PA was assessed at baseline, six months (post-intervention) and 12 months (maintenance phase) using the 7-Day Physical Activity Recall Interview. Costs were calculated from a payer perspective, and included personnel time (wage, fringe, and overhead), materials, equipment, software, and postage costs.

**Results:**

At six months, the PA intervention cost $29/person/month, compared to $15/person/month for wellness control. These costs fell to $17 and $9 at 12 months, respectively. Intervention participants increased their PA by an average of 72 min/week at six months and 94 min/week at 12 months, while wellness control participants increased their PA by an average of 30 min/week and 40 min/week, respectively. At six months, each minute increase in PA cost $0.18 in the intervention group compared to $0.23 in wellness control, which fell to $0.07 and $0.08 at 12 months, respectively. The incremental cost per increase in physical activity associated with the intervention was $0.15 at 6 months and $0.05 at 12 months.

**Conclusions:**

While the intervention was more costly than the wellness control, costs per minute of increase in PA were lower in the intervention. The print-based mail-delivered format has potential for broad, cost-effective dissemination, which could help address disparities in this at-risk population.

**Trial registration:**

NCT01583140; Date of Registration: 03/06/2012; Funding Source of Trial: National Institute of Nursing Research (NINR); Name of Institutional Review Board: Brown University IRB; Date of Approval: 05/19/2009.

## Background

Despite the well-documented benefits of physical activity, few Americans are regularly physically active. Recent studies show only approximately one-third (35 %) of Americans report engaging in regular physical activity [1] and even fewer are active for the recommended 30 min per day [2]. Concordantly, rates of conditions related to an inactive lifestyle, such as cardiovascular disease (CVD) and diabetes – and costs of treating these conditions – are high, and rising. The cost of treating diabetes alone accounts for more than 20 % of health care expenditures in the U.S., an increase of 41 % over the past five years, and it is estimated that the number of individuals with diagnosed diabetes will double by 2050 [3]. Costs of treating CVD are expected to triple by 2030, when 40.5 % of the population is projected to have some sort of CVD [4]. Developing widespread, cost-effective interventions to increase physical activity is essential given these projected trends in lifestyle-related conditions.

Of particular importance is developing interventions targeted to racial and ethnic minorities, who report less leisure time physical activity than non-Latino Whites and experience marked disparities in lifestyle-related conditions [1]. Rates of type II diabetes in Latinos, for example, are approximately double those of non-Latino Whites [5], and it is estimated that more than 50 % of Latinas born in this century will eventually develop diabetes [3]. Moreover, 42 % of Latino adults are obese compared with 32.6 % of Whites [6]. Latino women report particularly low rates of physical activity and are less likely to report meeting the national physical activity guidelines (>150 min of at least moderate activity/week, 38.2 %) than Non-Hispanic white women (50.9 %) or Latino men (47.0 %) [7]. Such health disparities are particularly concerning and likely related to the numerous linguistic, socioeconomic, and cultural barriers to quality medical care and public health intervention reported by Latinos, especially among Latino women [8].

To address these disparities, researchers have developed interventions targeting moderate-to-vigorous physical activity (MVPA) in Latinas, the success of which has been mixed. Some of these programs have shown promising increases in MVPA; however, many of these are face-to-face and/or site-based programs [9–11]. Such designs may be cost-prohibitive, could limit widespread dissemination, and do not address barriers commonly cited by Latinas, such as limited transportation and childcare duties. Home-based programs do not require frequent clinic visits, can be completed at a time and place convenient to the participant (e.g., at home when children are sleeping or occupied, at the park with family members), and may therefore be an especially appropriate, cost effective approach for this population.

We have developed a theory-driven, individually tailored, print-based mail-delivered MVPA intervention, which has been tested and shown to effectively increase MVPA in mostly non-Latino White populations. Through a series of formative research, cognitive interviews, focus groups, and pilot studies, this intervention was modified specifically for Latinas [12] and recently tested in a fully powered randomized controlled trial. Findings indicated significantly greater increases in MVPA in the intervention arm at six months [13] and again at 12-month follow-up compared to the control arm [14]. However, to date there are no cost-effectiveness analyses of such home-based physical activity interventions for Latinas. Therefore, the purpose of this paper is to assess the costs and cost-effectiveness of this linguistically and culturally adapted print-based intervention for Latinas.

## Methods

### Design

The Seamos Saludables study was a randomized controlled trial to test the efficacy of a Spanish-language print-based culturally and linguistically adapted MVPA intervention for Latinas compared to a wellness contact control. Intervention materials were individually tailored based on theoretical concepts of behavior change, and printed materials were delivered through the mail across a six-month intervention period followed by a tapered six-month maintenance period. Participants in the contact control group received Spanish language wellness materials delivered through the mail on the same schedule. Randomization was stratified by stage of motivational readiness for change to ensure participants in both conditions were equally ready to increase their PA.

### Setting & Sample

Participants were 266 women (age 18–65) who identified as Latina, who were free of chronic and/or serious health conditions, and were classified as underactive, getting less than 60 min of MVPA per week. Participation was limited to women for the current study, as rates of inactivity are higher among women than men in this community and the different physical activity behaviors and attitudes of Latino men merit independent study and an intervention that addresses their specific barriers and preferences [15]. Exclusion criteria included a BMI >45, current or planned pregnancy, and/or a history of coronary heart disease, diabetes, stroke, orthopedic problems, or other serious conditions that might make unsupervised MVPA unsafe, as determined by the Physical Activity Readiness Questionnaire [16]. Participants also had to be willing to be assigned to either arm of the trial. Detailed descriptions of inclusion/exclusion criteria, recruitment efforts, and flow diagram have been published previously [13]. Briefly, participants were healthy members of the community recruited via print and radio advertisements, community events, and flyers in local businesses with a high traffic of Latino clients. From this, 313 interested individuals were eligible for the study, of which 268 were randomized and included (21 could not be scheduled, 24 were deemed ineligible post-randomization). Participants (mean age = 40.6) were mostly (93 %) first generation, and generally low income with 69 % reporting an annual household income below $30,000.

Human subjects approval was granted by the Brown University Institutional Review Board.

### Intervention

The intervention was individually tailored based on the Transtheoretical Model (TTM, or Stages of Motivational Readiness for Change Model) [17] and Social Cognitive Theory (SCT) [18]. The TTM posits that individuals go through a series of motivational stages when changing behavior (precontemplation, contemplation, preparation, action, and maintenance), while SCT emphasizes the influence of cognitive, affective, and environmental influences on behavior. Participants filled out monthly surveys addressing their thoughts, feelings, and concerns about MVPA, and responses were entered into a computer expert system, which generated individually tailored reports. Participants also received regular tip sheets on topics related to MVPA (e.g., taking your pulse, social support). Detailed descriptions of the intervention protocol and materials have been published previously [12–14]. Briefly, participants received four mailings in the first month, two mailings in the second and third month, and one mailing in months four, five, and six (intensive intervention phase). After the main trial ended at six months, participants received booster doses of intervention materials at months 8, 10, and 12 (tapered maintenance phase). In addition to the mailings, participants engaged in a brief goal-setting session with research staff at baseline, received a phone call after one month to ask about current goals and progress, and participated in a repeat goal setting session at the six-month visit. They also received pedometers and were encouraged to monitor their PA by filling out monthly logs of minutes of MVPA and steps taken. Control participants received a short orientation and one-month call to ensure that both conditions received equal attention and number of contacts from staff.

### Wellness contact control condition

The wellness contact control (Wellness) received information on health topics other than PA mailed on the same schedule as the intervention. This included Spanish-language booklets developed by the National Heart, Lung, and Blood Institute (NHLBI) on heart-healthy behaviors. These were developed specifically for Latinos aged 18–54 with low levels of acculturation, education, and socio-economic status [19]. They also received tip sheets on other various wellness topics, and filled out monthly health assessment questionnaires on these topics to control for contact time and receive equal compensation.

### Measures

#### Clinical outcome measures

Physical activity was measured using the 7-Day Physical Activity Recall at baseline, 6 months and 12 months time points and was used to help estimate cost effectiveness (cost per additional minute of physical activity in the Intervention vs Wellness arms). The 7-Day PAR is an interviewer-administered self-report measure that quantifies weekly minutes of MVPA across settings, activity types, and intensities and was the study primary outcome. It has demonstrated acceptable reliability, internal consistency, and congruent validity with objective measures of PA, and also shows sensitivity to changes over time [20–25]. The main trial was powered on 7-Day PAR outcomes at six months based on findings from the pilot trial [13]. Follow-up data were also obtained at 12 months [14]. Aggregate minutes of MVPA over the study period were estimated by linearly interpolating the self-reported estimates at six and twelve months, subtracting baseline values. Because randomization effectively balanced PA between conditions at baseline, we did not further adjust for baseline PA values.

#### Costs

Costs were estimated from a payer perspective, excluding research and development costs. Activities to develop the intervention were extensive, and included translating all materials from a previously tested intervention, conducting focus groups to gather feedback from the target population, and further culturally adapting materials based on feedback and existing literature. Because the present analysis aims to estimate the costs of delivering the developed intervention, development costs are not reported. Costs included here focus instead on preparation (in this case, training), delivery (staff time and materials for the six-month intervention), and maintenance (tapered contact over the final six months).

We used the actual costs of delivering the intervention in the trial to estimate the costs of delivering the intervention in a real-world setting. Because the intervention is aimed at general wellness/prevention and requires no clinical training to deliver, it could be delivered in a variety of settings, such as a clinic/health system, wellness center, or fitness center. Costs included personnel time for training and for delivering the intervention, overhead, hardware/software, costs of the expert system, materials, printing, and postage. Costs were calculated at the time of the main outcome (six months), and at a later follow-up after a tapered maintenance phase (12 months). Because this analysis estimated the costs of implementing the developed intervention in a clinical or community setting, personnel time and materials devoted strictly to research activities (e.g. measuring PA, obtaining consent) were not included. Recruitment costs were also not included. Detailed per unit costs and unit numbers for the Intervention and Control conditions are shown in Tables 1 and 2, respectively.

**Table 1:** Unit quantities and costs for the intervention (*N* = 132)

		6 months			12 months (cumulative)		
	Unit cost	Units/person	Total units	Total cost	Units/person	Total units	Total cost
Staffing							
Trainer^a^	$47.59/h		4 h	$190		4 h	$190
Research assistant^a^	$30.88/h		314 h	$9704		380 h	$11,742
Materials							
Pedometers	$12.50	1	132	$1650	1	132	$1650
Intervention mailing (tip sheets, tailored report, envelopes, labels, printer ink)	$0.44	11	1452	$639	14	1848	$813
Scanner paper	$0.04	30	3950	$158	48	5060	$253
Orientation binders	$5.78	1	132	$763	1	132	$763
Stage-matched manuals	$1	6	792	$792	9	1188	$1188
Postage							
Questionnaires (w/return postage)	$2.50	5	660	$1650	8	1056	$2640
Intervention materials	$1.51	11	1452	$2193	14	1848	$2790

^a^Hourly costs for staff include salary, fringe benefits, and 10 % overhead. Salary amounts were sourced from standard university pay scales for a bachelor’s degree-level research assistant and master’s/doctoral-level trainer, including standard (44 %) benefits. Supply costs were based on wholesale office supply prices used to deliver the intervention. Postage costs were based on First Class mail with return postage for questionnaires

**Table 2:** Unit quantities and costs for the wellness control (*N* = 134)

		6 months			12 months (cumulative)	12 months (cumulative)	12 months (cumulative)
	Unit cost	Units/person	Total units	Total cost	Units/person	Total units	Total cost
Staffing							
Trainer^a^	$47.59/h		2 h	$95		4 h	$95
Research assistant^a^	$30.88/h		156 h	$4821		183 h	$5649
Materials							
Wellness mailing (tip sheets, envelopes, labels, printer ink)	$0.43	11	1474	$635	14	1876	$808
Scanner paper	$0.04	24	3216	$129	30	4020	$161
Orientation binders	$5.18	1	134	$694	1	134	$694
Wellness booklets	$1	6	804	$804	9	1206	$1206
Postage							
Questionnaires (w/ return postage)	$2.50	5	670	$1675	8	1072	$2680
Wellness materials	$1.51	11	1474	$2226	14	1876	$2833

^a^Hourly costs for staff include salary, fringe benefits, and 10 % overhead. Salary amounts were sourced from standard university pay scales for a bachelor’s degree-level research assistant and master’s/doctoral-level trainer, including standard (44 %) benefits. Supply costs were based on wholesale office supply prices used to deliver the intervention. Postage costs were based on First Class mail with return postage for questionnaires

#### Personnel costs

Staff times were calculated by totaling the time required for each step of delivering the intervention or wellness materials. For the intervention condition, this included scanning questionnaires (which are needed to generate tailored reports), generating and printing tailored reports, mailing reports, compiling orientation materials, and conducting initial orientation/goal setting sessions and one-month follow-up calls. For the Wellness condition, this included printing and mailing materials, compiling orientation materials, and conducting initial orientation sessions and one-month calls. Time needed to train staff members to print materials, conduct orientation sessions and make one-month calls for each condition (this included costs for trainee and trainer) were considered, along with factors such as time needed to contact participants, including failed contact attempts. Time devoted to each task was determined by asking experienced research staff to provide a range for the time needed for a specific task, and to report how many could be completed within one hour to verify the range. Because staff became more efficient throughout the intervention, we used the midpoint estimate for each task.

To assess personnel costs of delivering the intervention, we multiplied the time devoted to intervention activities by standard salary and fringe rates for individuals with the proper education level to deliver the intervention using 2014 rates. Salary rates were sourced from standard university pay scales for a research assistant with a bachelor’s degree to deliver the intervention (annual salary of $38,941, 44 % benefits, total of $56,153 or $28.08/h) and for a masters/doctoral-level individual to provide necessary training (annual salary of $60,000, 44 % benefits, total of $86,520 or $43.26/h). All of these rates were increased by 10 % to include overhead costs for shared facilities use.

#### Computer expert system

The computer expert system was used to generate individually tailored feedback reports based on progress and responses to psychosocial questionnaires and select PA manuals matched to the intervention participant’s current stage of readiness for PA. Costs for the expert system include hardware and software costs, materials costs, and mailing costs. Hardware costs include a computer, printer, and an optical scanner used to read responses to questionnaires and input them into the expert system. Hardware costs were estimated using market prices in June 2014. Software costs include scanner software and costs for updating the expert system software. Scanner software was estimated using its market price and the cost of updating the expert system was estimated using the resources required to install and adapt the software to a new computer and a specific setting. Hardware and scanner software costs were depreciated using the straight-line method of depreciation, assuming three years of use during the study and a five-year depreciation period.

Costs of the computer and printer were included for both the Intervention and Wellness groups, while the scanner, scanner software, and expert system software were only included for the Intervention group.

#### Materials & Printing

Material costs were calculated using standard wholesale office supply figures in 2014. Printing and binding costs for motivationally matched manuals were $1 per booklet. Spanish-language wellness booklets for the Wellness group can be downloaded from the National Heart, Lung, and Blood Institute (NHLBI), and were also estimated at $1 per booklet for printing and binding. Paper costs were also calculated per page for tip sheets, printed reports, and questionnaires. Participants in both conditions also received a binder at randomization with tip sheets, contact information, and (for the intervention group) a CD for exercising. Costs for printing were estimated per page using market prices in 2014. Participants in the Intervention group also received pedometers to fill out monthly logs of steps taken, at a cost of approximately $12.50 each.

#### Postage

Postage for intervention and wellness materials was estimated at $1.51 per mailing using First Class mail. Questionnaires cost $2.50 per mailing, which included postage-paid envelopes for returning completed questionnaires. The Wellness and Intervention conditions were controlled for number of contacts, and thus received the same number of mailings.

### Analysis

Total costs were determined by summing the cost of materials, equipment, software, personnel time, and postage for each condition. Total minutes of MVPA over the course of the study were calculated by performing linear interpolation from baseline to six month and then six month to 12-month visits. Cost effectiveness was determined by dividing the total cost per person at each visit by the total minutes of activity performed per person through six and 12 months. Incremental costs for the intervention vs. control groups were then calculated by dividing the difference in total minutes of MVPA between the Intervention and Wellness groups by the difference in total costs. Sensitivity analyses were then computed using a 20 % increase and 20 % decrease in costs for personnel, software, hardware, and for clinical outcomes (physical activity) to generate a range of uncertainty for incremental costs.

## Results

### Clinical outcome measures

As described elsewhere [13], Intervention participants (*n* = 132) increased their self-reported MVPA from a mean of 1.87 min/week (SD = 6.86) at baseline to 73.36 min/week (SD = 89.73) at six months, whereas Wellness participants (*n* = 134) increased their MVPA from a mean of 3.02 min/week (SD = 10.30) at baseline to 32.98 min/week (SD = 82.82) at six months (*p* < .05). These physical activity gains were maintained at 12 months [14]. On average, Intervention participants reported 95.79 min/week (SD = 114.89) of MVPA at 12 months, compared to 43.42 min/week (SD = 88.75) for Wellness participants.

Linear interpolation was used to estimate the increase in MVPA over six and twelve months for the Intervention and Wellness control group. Assuming a linear increase in MVPA, intervention participants increased their MVPA by a total of 929 min over the course of the six-month intervention, while Wellness participants increase their MVPA by a total of 389 min. At follow-up, Intervention participants increased their MVPA by a total of 3080 min from baseline to 12 months, and Wellness participants by 1304 min.

### Costs

The value of each cost component appears in Table 3 (6 months) and Table 4 (12 months). Total cost of the intervention at 6 month was $22,673 or $29 per participant per month, while the total cost of the Wellness control was $11,905 or $15 per person per month. At 12 months, the total cost of the intervention was $26,963 or $17 per participant per month, while the total cost of the Wellness control was $14,786 or $9 per person per month.

**Table 3:** Components of the 6-month cost estimate by intervention arm

	Seamos saludables intervention	Wellness control
	*N* = 132	*N* = 134
Personnel^a^		
Research assistant		
Training	$314	$157
Orientation	$3058	$1035
One-month calls	$679	$483
Preparing and mailing materials	$5436	$3241
Computer expert system		
Hardware		
Computer	$420	$420
Printer	$240	$240
Scanner	$2004	
Software		
Scanner	$870	
Updating the expert system	$1400	
Material and mailing costs		
Materials		
Pedometers	$1650	
Paper, printing and binding	$2352	$2262
Mailing	$3843	$3901
Total costs	$22,673	$11,905
Average cost per participant	$172	$89
Average cost per participant per month	$29	$15

^a^Personnel costs were calculated by multiplying total time spent in each activity by standard hourly rates for a research assistant and/or supervisor with the appropriate qualifications for each task. Material costs were based on wholesale office supply costs for materials used per participant. Postage costs were based on First Class mail with return postage for questionnaires

**Table 4:** Components of the 12-month cost estimate by intervention arm

	Seamos saludables intervention	Wellness control
	*N* = 132	*N* = 134
Personnel		
Research assistant		
Training	$314	$157
Orientation	$3057	$1035
One-Month calls	$679	$483
Preparing and mailing materials	$7881	$4070
Computer expert system		
Hardware		
Computer	$420	$420
Printer	$240	$240
Scanner	$2004	
Software		
Scanner	$870	
Updating the expert system	$1400	
Material and mailing costs		
Materials		
Pedometers	$1650	
Printing and binding	$3017	$2869
Mailing	$5430	$5513
Total costs	$26,963	$14,786
Average cost per participant	$204	$110
Average cost per participant per month	$17	$9

^a^Personnel costs were calculated by multiplying total time spent in each activity by standard hourly rates for a research assistant and/or supervisor with the appropriate qualifications for each task. Material costs were based on wholesale office supply costs for materials used per participant. Postage costs were based on First Class mail with return postage for questionnaires

### Cost effectiveness

Table 5 provides estimates of costs per minute for increases in physical activity in the Intervention and Wellness arms. The cost per minute increase in MVPA at 6 months was $0.18 in the Intervention group and $0.23 in the Wellness control. The cost per minute at 12 months was $.07 in the Intervention and $.08 in the Wellness control. The incremental cost per minute of the intervention relative to Wellness control was $0.15 per minute at 6 months and $0.05 per minute at 12 months.

**Table 5:** Costs per minute increase in physical activity

	Seamos saludables intervention	Wellness control
Increase in MVPA		
Total per person minutes of MVPA from baseline to 6 months	929	389
Total per person minutes of MVPA from baseline to 12 months	3080	1304
Minutes of MVPA per person per month at 6 months	155	65
Minutes of MVPA per person per month at 12 months	256	109
Cost per minute of increase in MVPA		
Costs per minute increase in MVPA at 6 months	$0.18	$0.23
Costs per minute increase in MVPA at 12 months	$0.07	$0.08
Incremental cost per minute of increase in MVPA between the intervention and wellness control		
Incremental costs per minute at 6 months	$0.15	n/a
Incremental costs per minute at 12 months	$0.05	n/a

^a^Total minutes of activity were calculated using linear interpolation of mean minutes at baseline to six-month follow-up, and from six-month follow-up to 12-month follow-up. Costs per minute increase in PA are based on mean PA increases and aggregate costs for each condition. Incremental costs represent the additional cost per minute of activity for Intervention participants beyond those reported by the control group

Sensitivity analyses are shown in Table 6. Increasing or decreasing personnel costs by 20 % changed incremental costs only slightly, and there was virtually no change in incremental costs when the expert system maintenance and hardware costs were adjusted by 20 % in both directions. The largest difference was seen when intervention outcomes (physical activity) were adjusted up or down by 20 %, in which case 6-month incremental costs ranged from $0.13 to $0.19 per minute, respectively, and 12-month incremental costs ranged from $0.04 to $0.07 per minute, respectively.

**Table 6:** Sensitivity analyses for incremental costs per minute

	6-month Incremental Cost per Minute	12-month Incremental Cost per Minute
Standard calculation	$0.15	$0.05
Staffing costs		
+ 20 %	$0.17	$0.06
− 20 %	$0.14	$0.05
Intervention effectiveness		
+ 20 %	$0.13	$0.04
− 20 %	$0.19	$0.07
Expert system maintenance		
+ 20 %	$0.16	$0.05
− 20 %	$0.15	$0.05
Hardware costs		
+ 20 %	$0.16	$0.05
− 20 %	$0.15	$0.05

## Discussion

The current study showed that while the intervention was more costly to deliver than the contact control, costs per minute of increase in PA were lower in the intervention group. Cost of the intervention was approximately double that of delivering the Wellness control materials; however, because increases in MVPA in the Intervention group were more than double those of the Wellness group, analyses show that the Intervention was more cost effective than the Wellness condition. The cost of increasing MVPA was $0.18 per minute for Intervention participants at six months, compared to $0.23 per minute for Wellness. MVPA increased in both groups over the maintenance phase from six to 12 months despite limited additional intervention content or contact with study staff, thus costs over the whole 12 months were considerably lower ($0.07 and $0.08 per minute, respectively). Cost of maintaining the intervention effect, then, was quite low, and increased cost-effectiveness overall.

In the current study, the Intervention and Wellness groups were matched for contact, thus costs were essentially equal for materials and postage. The majority of the extra cost in the intervention group came from the extra equipment (scanner and software) and extra staff time required to produce the individually tailored reports, and in providing pedometers for self-monitoring. These additional intervention components appeared to be worth the added cost as the intervention condition increased their activity significantly more than the Wellness group. However, these analyses must be interpreted with caution, as the purpose of the control group was to match for contact time and not to compare the intervention to existing MVPA materials, thus the wellness materials did not contain any MVPA information. It is possible a less intensive physical activity content-only intervention might produce some change in physical activity behavior at a lower cost.

The costs reported here reflect the actual costs incurred to deliver the intervention, and so act as the best estimate of the costs of delivering the intervention in a real-world setting. The format of the intervention allows for great flexibility in delivery setting, and while this is an asset of the intervention, there could be marked variability in costs and cost-effectiveness depending on the delivery setting and the target population. Staff salaries within a health system, for example, may be higher than in a wellness center or fitness program, while the effectiveness of the intervention and need for additional follow-up may vary across target populations. The time and costs associated with preparing the intervention for delivery could also vary depending on infrastructure, access to hardware and supplies, and existing staffing, and it is likely the cost of preparation would decrease markedly over time. In this respect, the costs here may best be seen as a guide to be tailored to specific payer systems rather than an exact estimate across settings.

Comparing the costs of delivering this MVPA intervention to those found in past studies in this area is difficult, given the wide variety of physical activity outcomes (minutes/week of MVPA, kcals, percent meeting guidelines) and analytical approaches found in the literature. For example, our analyses focused on estimating the costs of implementing the developed intervention in an outside setting, such as a clinic or wellness center, and included factors such as personnel time (salary, benefits, and overhead) for training and for delivering the intervention, hardware/software, costs of the expert system, materials, printing, and postage. Thus, we are not able to compare our findings with those studies that approached cost effectiveness analyses from a broader health care perspective, focusing on future health care costs and quality adjusted life years gained [26] and/or societal perspective, including intervention costs, health care costs, participant and family costs and productivity losses [27]. Moreover the studies that have taken a similar clinic-focused approach to cost effectiveness included different variables in their analyses (e.g. recruitment costs) than the current study [28].

Most research on the costs of PA interventions has been conducted on face-to-face interventions and/or in non-Hispanic samples [29–31]. We were unable to locate any published cost analyses of PA interventions conducted among Latinas, despite a thorough literature review. The costs of delivering this tailored print intervention in the current study with Latinos were lower than those found in similar past print-based intervention studies conducted among mostly Non-Hispanic White samples ($204 per person at 12 months [28] vs. $480 and $429.69 at 12 months [32]). Such differences may be partially explained by the exclusion of recruitment and exclusive use facility costs from our analyses, given the payer perspective of the current study. Moreover, there have likely been improvements in efficiency due to staff training/experience over the years, which could have positively influenced cost effectiveness in the current, most recent print-based study from this line of research.

The use of mediated (i.e., non face-to-face) delivery channels has the potential to increase reach without greatly increasing costs. Cost per participant for the current intervention would likely decrease even further with a larger sample size, as equipment needed to generate tailored reports (i.e., computer, scanner, and software) are fixed costs that would remain constant regardless of the number of participants. Interventions relying almost entirely on fixed cost delivery channels, such as texting and Internet-based interventions, may therefore be especially cost effective when broadly distributed. One analysis of a print-based vs. Internet-based PA intervention in mostly Non-Hispanic Whites found that the Internet-based intervention cost more than three times as much per participant ($122.52/month vs. $35.81/month), largely due to the high start up cost [32] A break-even analysis, however, revealed that the Internet intervention became more cost-efficient per participant, relative to the print intervention, when the total number of participants exceeded 352, and cost per participant continued to decrease with larger samples [32] Thus, it may be more cost efficient to use the Internet rather than mail delivery for wide-scale dissemination in this rapidly growing target population. Moreover, this would allow for automation and distance delivery of goal setting and research assessments currently being conducted face-to-face by research staff and further reduce costs. However, data suggest that Internet access is still low (<50 %) in Latinos with lower levels of acculturation and education [33], thus a print-based mail-delivered intervention is still a useful, cost effective way to intervene on this high risk population on a broad scale.

There are many strengths to the current study including the use of a randomized controlled trial research design, and reliance on current market data for materials, equipment, and personnel cost. Moreover, we recruited a diverse sample of Latinas. Most were from the Dominican Republic, Colombia, and Puerto Rico, subgroups which are often underrepresented in research with Latinos. Limitations include the reliance on self report for the staff time sampling exercise, which may have resulted in a biased account of time spent on completing the intervention related tasks. Also, physical activity was measured via a subjective measure (the 7-Day PAR), which is significantly correlated with objective measures [20] but may still be subject to bias. This cost effectiveness analysis was based on a single study RCT, and though some costs were fixed (e.g., number of booklets, postage) others, as emphasized above, could vary more with different settings or other populations, as could physical activity outcomes. Because this was a short-term, single study design, we were also unable to project long-term costs, such as health care savings from maintained physical activity. Finally, we did not include recruitment costs, as those associated with the trial would not be representative of those in a non-research setting, though some recruitment costs may be necessary in implementing the intervention in a community or clinic setting.

Given the marked disparities in chronic disease in Latinas and the high costs of treating these diseases, the clear next step is to determine whether such interventions could translate into reduced disparities and health care cost savings. While the current study focused on relatively short-term PA outcomes, future studies with Latinas could collect additional data and use computer simulation models to extrapolate results to 5-year, 10-year and lifetime horizons for health effects and costs and quality-adjusted life years, similar to methods used by Peels et al. in Dutch adults [34]. For all extrapolated time horizons in those analyses, the print and Web-based interventions produced decreases in incidence of diabetes, colon cancer, breast cancer, acute myocardial infarctions, and stroke and increased quality-adjusted life years, as a result of increased PA. Such data supporting the long-term cost-effectiveness of tailored PA interventions for Latinos, and potential for chronic disease impact may encourage large-scale implementation of these interventions, thereby reducing health disparities and benefiting public health.

## Conclusion

Results from the current study indicated that tailored print materials are a cost-effective approach to increasing physical activity among Latinos. Thus this study replicates past findings in this area (in mostly non-Hispanic, white samples in the US; Dutch adults aged over fifty) and extends this line of research to a new at risk target population, Latinas. Future directions will include exploring Internet-based interventions given their potential to reach a large number of sedentary individuals and recent increases in Internet access/use among Latinos.

## References

[1] Schiller JS, Lucas JW, Ward BW, Peregoy JA (2012). Summary health statistics for U.S. adults: National Health Interview Survey, 2010. Vital Health Statistics.

[2] Troiano RP, Berrigan D, Dodd KW, Masse LC, Tilert T, McDowell M (2008). Physical activity in the United States measured by accelerometer. Med Sci Sports Exerc.

[3] Narayan KM, Boyle JP, Geiss LS, Saaddine JB, Thompson TJ (2003). Lifetime risk for diabetes mellitus in the United States. JAMA.

[4] Heidenreich PA, Trogdon JG, Khavjou OA, Butler J, Dracup K, Ezekowitz MD (2011). Forecasting the future of cardiovascular disease in the United States: a policy statement from the American Heart Association. Circulation.

[5] Cowie CC, Rust KF, Ford ES, Eberhardt MS, Byrd-Holt DD, Li C (2009). Full accounting of diabetes and pre-diabetes in the U.S. population in 1988–1994 and 2005–2006. Diabetes Care.

[6] Ogden CL, Carroll MD, Kit BK, Flegal KM (2013). Prevalence of obesity among adults: United States, 2011–2012. NCHS data brief, no. 131.

[7] Blackwell DL, Lucas JW, Clarke TC (2014). Summary health statistics for U.S. adults: National Health Interview Survey: National Center for Health Statistics. Vital Health Stat.

[8] Cusi K, Ocampo GL (2011). Unmet needs in Hispanic/Latino patients with type 2 diabetes mellitus. Am J Med.

[9] Hovell MF, Mulvihill MM, Buono MJ, Liles S, Schade DH, Washington TA (2008). Culturally tailored aerobic exercise intervention for low-income Latinas. Am J Health Promot.

[10] Ayala GX (2011). Effects of a promotor-based intervention to promote physical activity: Familias Sanas y Activas. Am J Public Health.

[11] Eakin EG, Bull SS, Riley KM, Reeves MM, McLaughlin P, Gutierrez S (2007). Resources for health: a primary-care-based diet and physical activity intervention targeting urban Latinos with multiple chronic conditions. Health Psychol.

[12] Pekmezi DW, Neighbors CJ, Lee CS, Gans KM, Bock BC, Morrow KM (2009). A culturally adapted physical activity intervention for Latinas: a randomized controlled trial. Am J Prev Med.

[13] Marcus BH, Dunsiger SI, Pekmezi DW, Larsen BA, Bock BC, Gans KM (2013). THE SEAMOS SALUDABLES Study: a Randomized Controlled Physical Activity Trial Of LATINAS. Am J Prev Med.

[14] Marcus BH, Dunsiger SI, Pekmezi D, et al. Twelve-Month Physical Activity Outcomes in Latinas in the Seamos Saludables Trial. Am J Prev Med. 2014;26:392-400. 10.1016/j.amepre.2014.08.032 PMC5718346 25442225

[15] Larsen BA, Dunsiger SI, Hartman S, Nodora J, Pekmezi DW, Marquez B, et al. Activo: Assessing the feasibility of designing and implementing a physical activity intervention for Latino men. International Journal Men’s Health. 2014;13(1)60-71.

[16] American College of Sports Medicine (2009). ACSM’s guidelines for exercise testing and prescription.

[17] Prochaska JO, DiClemente CC (1986). Toward a comprehensive model of change.

[18] Bandura A (1986). SOCIAL Foundations Of Thought And Action: a Social Cognitive Theory.

[19] National Heart Lung and Blood Institute. Risk factor book for Latinos. U.S. Department of Health and Human Services. http://www.nhlbi.nih.gov/health/resources/heart/latino-package. 2008.

[20] Prince SA, Adamo KB, Hamel ME, Hardt J, Connor Gorber S, Tremblay M (2008). A comparison of direct versus self-report measures for assessing physical activity in adults: a systematic review. Int J Behav Nutr Phys Act.

[21] Hayden-Wade HA, Coleman KJ, Sallis JF, Armstrong C (2003). Validation of the telephone and in-person interview versions of the 7-day PAR. Med Sci Sports Exerc.

[22] Leenders NY, Sherman WM, Nagaraja HN, Kien CL (2001). Evaluation of methods to assess physical activity in free-living conditions. Med Sci Sports Exerc.

[23] Dunn AL, Garcia ME, Marcus BH, Kampert JB, Kohl HW, Blair SN (1998). Six-month physical activity and fitness changes in Project Active, a randomized trial. Med Sci Sports Exerc.

[24] Dunn AL, Marcus BH, Kampert JB, Garcia ME, Kohl HW, Blair SN (1999). Comparison of lifestyle and structured interventions to increase physical activity and cardiorespiratory fitness: a randomized trial. JAMA.

[25] Rauh MJ, Hovell MF, Hofstetter CR, Sallis JF, Gleghorn A (1992). Reliability and validity of self-reported physical activity in Latinos. Int J Epidemiol.

[26] Over EA, Wendel-Vos GW, van den Berg M, Reenen HH, Tariq L, Hoogenveen RT, et al. Cost-effectiveness of counseling and pedometer use to increase physical activity in the Netherlands: a modeling study. Cost Resour Alloc. 2012;10(1):13. 10.1186/1478-7547-10-13 PMC3495195 23006466

[27] Golsteijn R, Peels DA, Evers S, Bolman C, Mudde AN, de Vries H, et al. Cost-effectiveness and cost-utility of a Web-based or print-delivered tailored intervention to promote physical activity among adults aged over fifty: an economic evaluation of the Active Plus intervention. Int J Behav Nutr Phys Act. 2014;11(1):122. 10.1186/s12966-014-0122-z PMC4189727 25262435

[28] Sevick MA, Napolitano MA, Papandonatos GD, Gordon AJ, Reiser LM, Marcus BH (2007). Cost-effectiveness of alternative approaches for motivating activity in sedentary adults: results of Project STRIDE. Prev Med.

[29] Proper KI, de Bruyne MC, Hildebrandt VH, van der Beek AJ, Meerding WJ, van Mechelen W (2004). Costs, benefits and effectiveness of worksite physical activity counseling from the employer’s perspective. Scand J Work Environ Health.

[30] Sevick MA, Dunn AL, Morrow MS, Marcus BH, Chen GJ, Blair SN (2000). Cost-effectiveness of lifestyle and structured exercise interventions in sedentary adults: results of project ACTIVE. Am J Prev Med.

[31] Stevens W, Hillsdon M, Thorogood M, McArdle D (1998). Cost-effectiveness of a primary care based physical activity intervention in 45–74 year old men and women: a randomised controlled trial. Br J Sports Med.

[32] Lewis BA, Williams DM, Neighbors CJ, Jakicic JM, Marcus BH (2010). Cost Analysis of Internet vs. Print Interventions for Physical Activity Promotion. Psychol Sport Exerc.

[33] Lopez MH, Gonzalez-Barrera A, Patten E. Closing the digital divide: Latinos and technology adoption. Pew Research Center. 2013.

[34] Peels D, Hoogenveen R, Feenstra T, Golsteijn R, Bolman C, Mudde AN, et al. Long-term health outcomes and cost-effectiveness of a computer-tailored physical activity intervention among people aged over fifty: modelling the results of a randomized controlled trial. BMC Public Health. 2014;14(1):1099. 10.1186/1471-2458-14-1099 PMC4221676 25342517

